# Supplementary material for: Inattentional blindness in anesthesiology: A gorilla is worth one thousand words
Source: PLoS One. 2021 Sep 23;16(9):e0257508. doi: 10.1371/journal.pone.0257508 (PMC8459955; doi:10.1371/journal.pone.0257508)
Supplement: S1 File — (PDF) [file pone.0257508.s001.pdf]

Management of a day-case surgery operating session

Dear colleagues,
here is a short survey that should take less than 5 minutes.
We propose a series of five hypothetical cases scheduled in a single operating session (day-case surgery)and you will be asked to choose the anesthesia plan for these five healthy patients.

All patients have no or mild health problems, but if you have any observation there will be space for your comment for each case.

The results of this survey will be reported in aggregate terms and potentially published. Please note, all answers will remain anonymous, and it will not be possible to identify your responses.

Thank you for taking part!

For any issue feel free to contact us
Alessandro De Cassai, M.D.
(alessandro.decassai@aopd.veneto.it)

*Required

1. Are you an anesthesiologist/anesthesiologist resident? *

   Mark only one oval.

   - ( ) Anesthesiologist
   - ( ) Anesthesiology resident
   - ( ) No

2. Country *

   ____

3. Please select the answer that best describes your institution type. *

Mark only one oval.

- ◯ Public Hospital: University/teaching
- ◯ Public Hospital: Non-teaching
- ◯ Private Hospital
- ◯ Other: ______________________

4. Please select the answer that indicates your experience in anesthesia *

Mark only one oval.

- ◯ Resident
- ◯ 0-5 years
- ◯ 6-10
- ◯ 11-15 years
- ◯ 16+years

Case 1 - Patient 1

A 35 years old patient presents for breast lumpectomy. She is:
-Healthy
-Non-smoking
-ASA-PS I

Preoperative EKG and Chest radiography are normal and below reported.

Chest Radiography - Patient 1

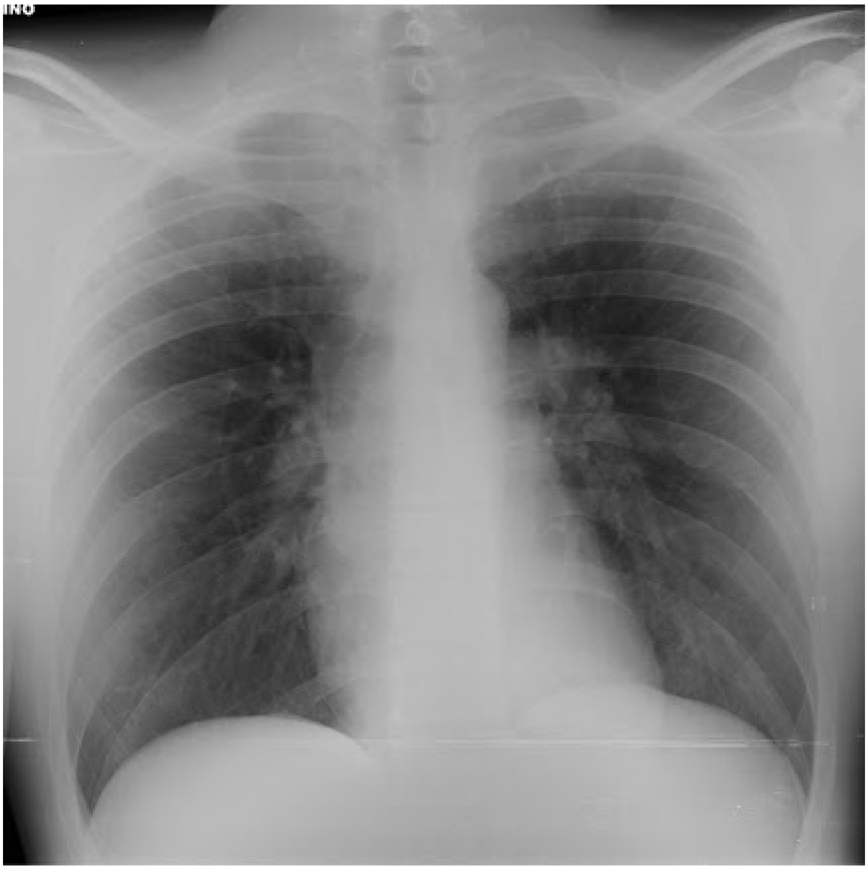

EKG - Patient 1

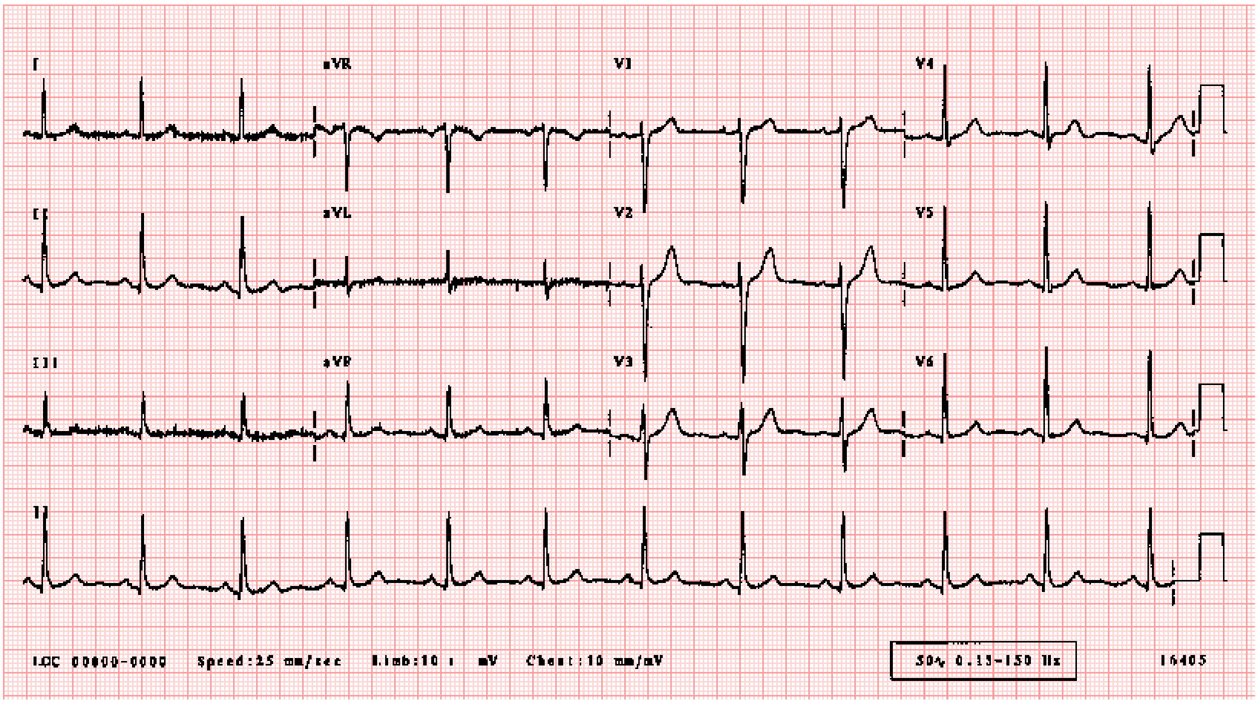

5. How would you manage the case?

Mark only one oval.

- ( ) General Anesthesia
- ( ) General Anesthesia + PECS
- ( ) General Anesthesia + ESP
- ( ) Sedation
- ( ) Other: ____________________

6. Do you need any other pre-operative examination? If yes, which one?

____

7. Any comment on Patient 1?

____

Case 2 - Patient 2

A 54 years old patient presents for malignant melanoma of the left leg skin exeresis, located on the lower outer thigh. She presents:
- Controlled hypertension (monotherapy with beta blocker)
- Asthma
- Dyslipidemia
- Non-smoking
- ASA-PS II

Preoperative laboratory findings are normal (in particular blood count, glycemia, sodium, potassium, coagulation and platelets count)
Preoperative EKG and Chest radiography are normal and below reported..

Chest Radiography - Patient 2

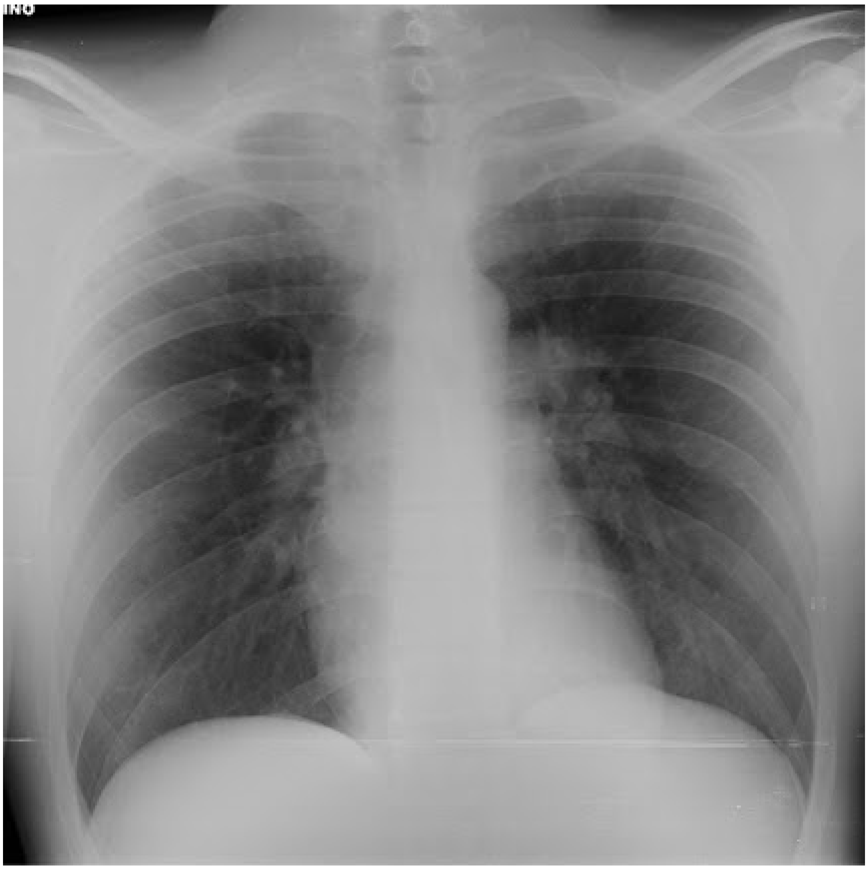

EKG - Patient 2

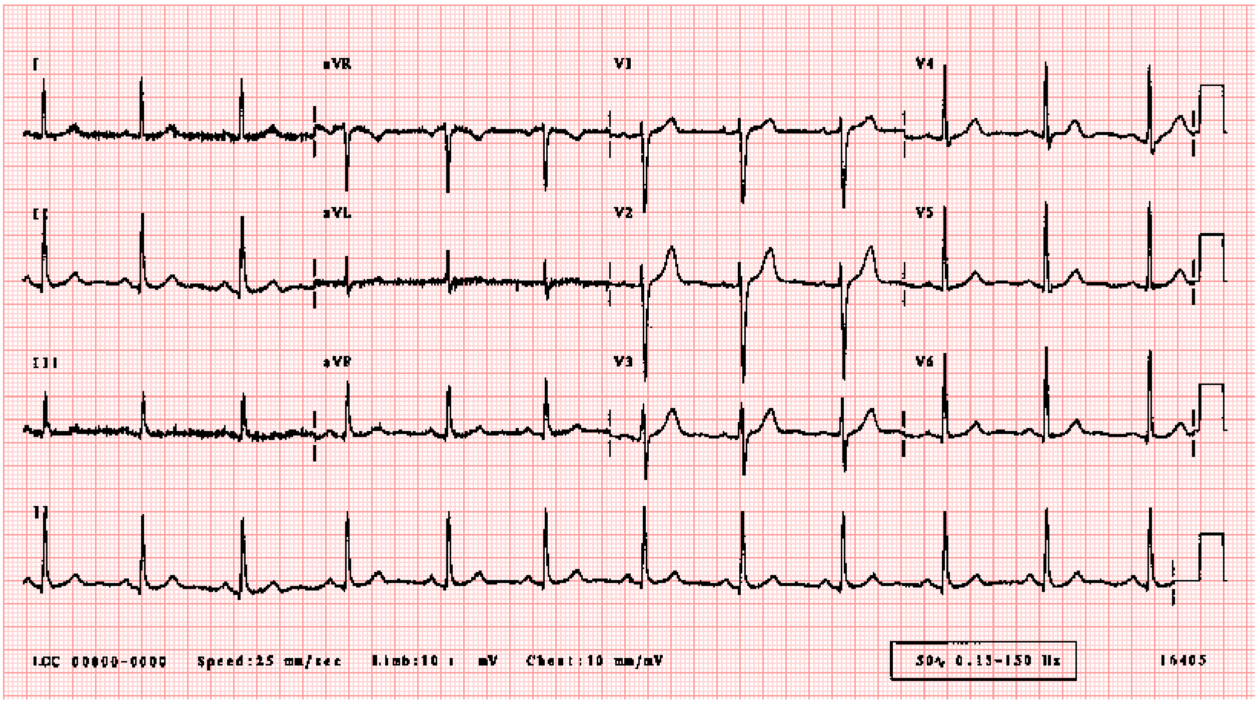

8. How would you manage the case?

Mark only one oval.

- General Anesthesia
- Spinal Anesthesia
- Sedation + Local Anesthesia
- Other: ____________________

9. Do you need any other pre-operative examination? If yes, which one?

____

10. Any comment on Patient 2?

____

Case 3- Patient 3

A 33 years old patient presents for small nevus (0,4 cm)resection of the left leg. He is:
-Healthy, anxious
-Mildly obese (BMI 32)
-Non-smoking
-ASA-PS II

Preoperative laboratory findings are normal (in particular blood count, glycemia, sodium, potassium, coagulation and platelets count)
Preoperative EKG and Chest radiography are normal and below reported..

Chest Radiography - Patient 3

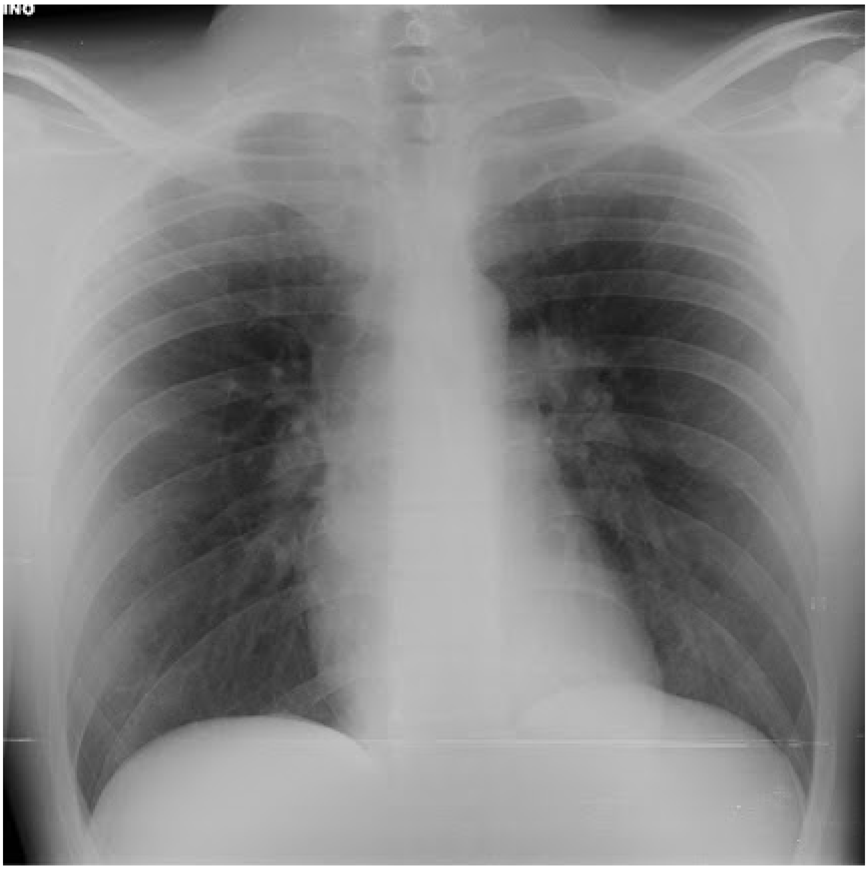

EKG - Patient 3

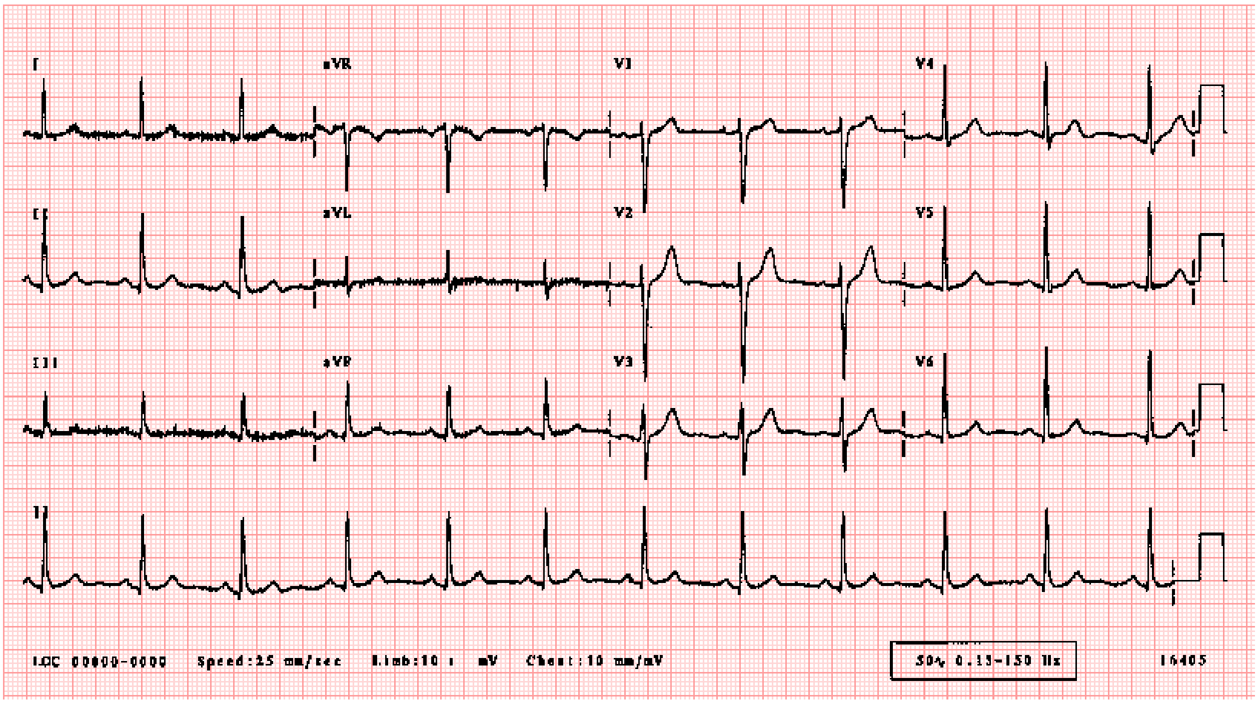

11. How would you manage the case?

Mark only one oval.

- General Anesthesia
- Spinal Anesthesia
- Sedation
- Other: ______________________

12. Do you need any other pre-operative examination? If yes, which one?

____

13. Any comment on Patient 3?

____

Case 4 - Patient 4

A 55 years old patient presents for incsion and drainage of sinus pilonidalis.
Medical history:
-Hypertension
-Smoking (2 pack/die)
-Aspirin 100 mg/daily (primary) prevention
-ASA-PS II

Preoperative laboratory findings are normal (in particular blood count, glycemia, sodium, potassium, coagulation and platelets count)
Preoperative EKG and Chest radiography are normal and below reported..

Chest Radiography - Patient 4

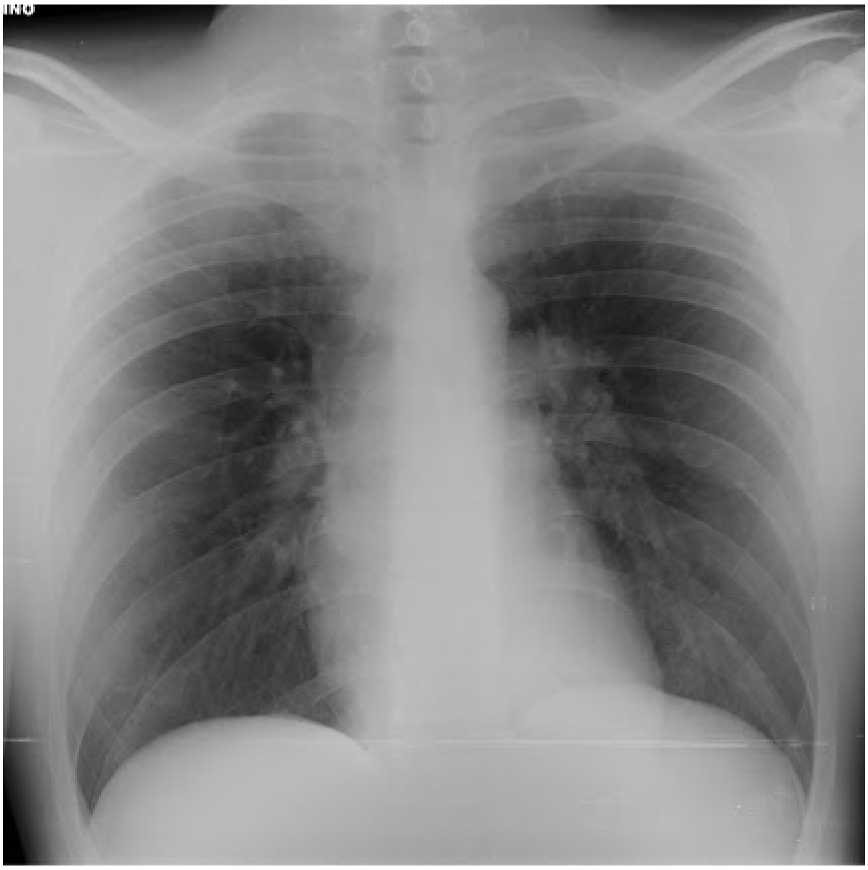

EKG - Patient 4

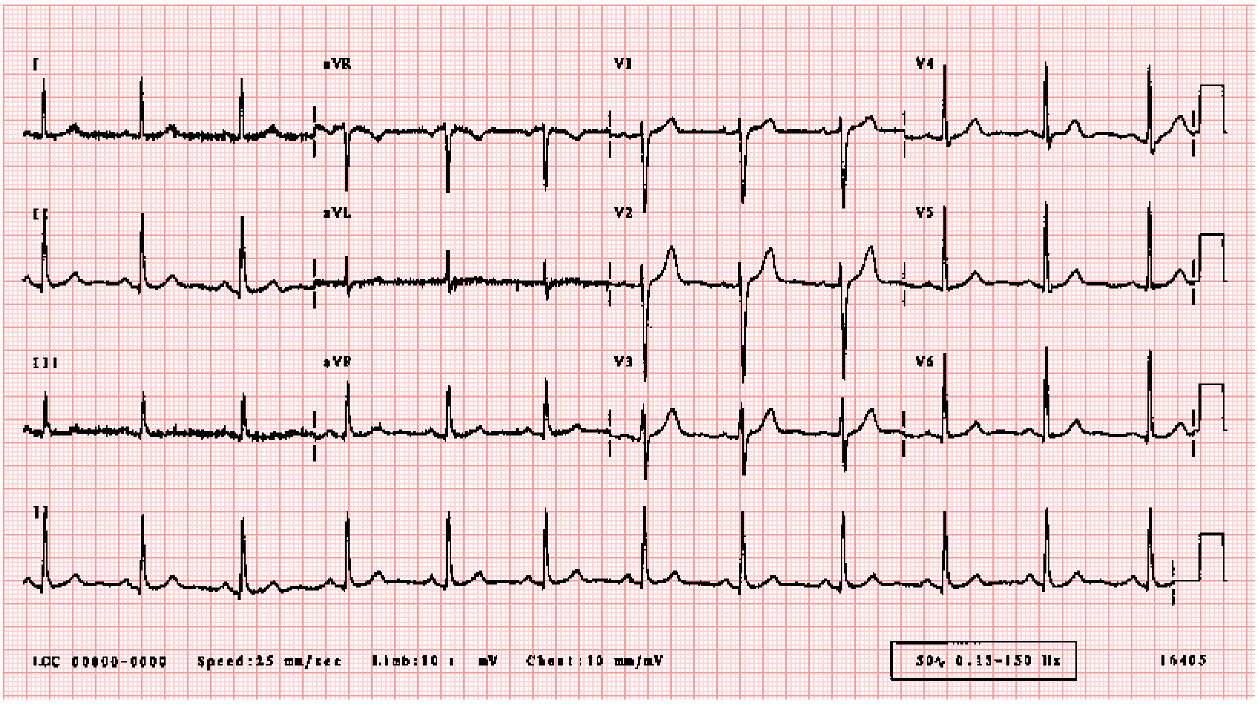

14. How would you manage the case?

Mark only one oval.

- ( ) General Anesthesia
- ( ) Spinal Anesthesia

15. Do you need any other pre-operative examination? If yes, which one?

____

16. Any comment on Patient 4?

____

Case 5 - Patient 5

A 30 years old patient presents for left inguinal hernia. Surgeon suggest a open inguinal hernia repair
-Healthy
-Weightlifter
-Non-smoking
-ASA-PS I
-No medications

Preoperative laboratory findings are normal (in particular blood count, glycemia, sodium, potassium, coagulation and platelets count)
Preoperative EKG and Chest radiography are normal and below reported..

Chest Radiography - Patient 5

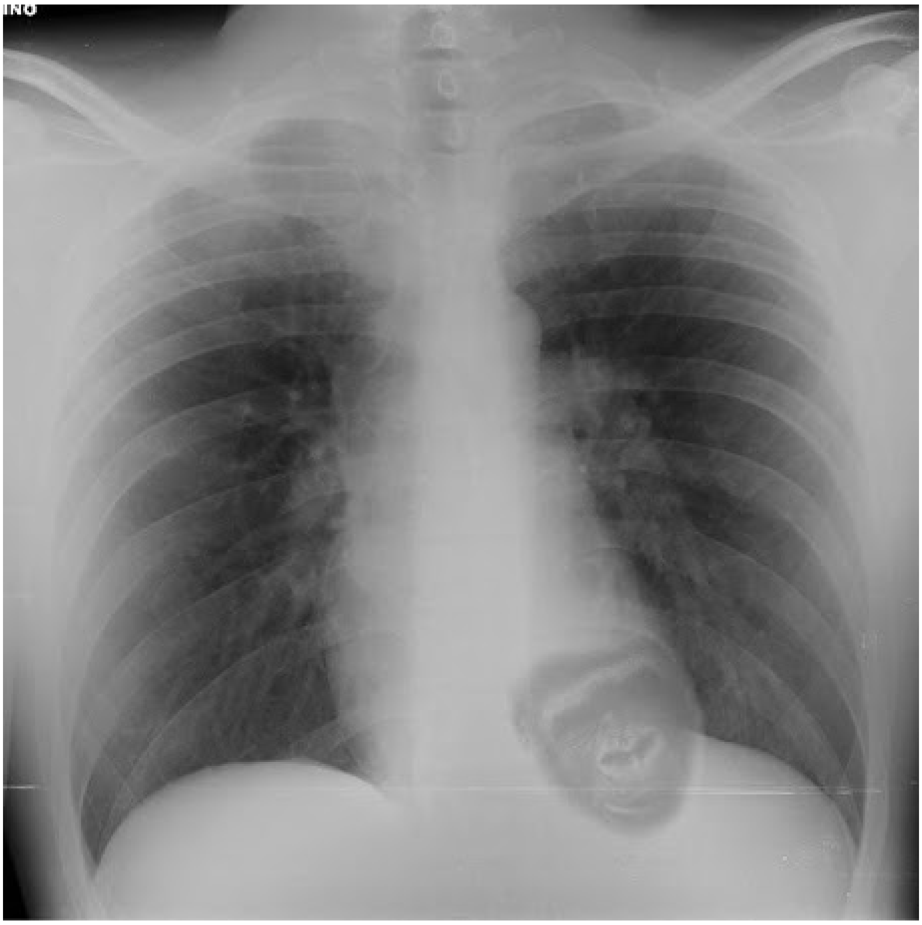

EKG - Patient 5

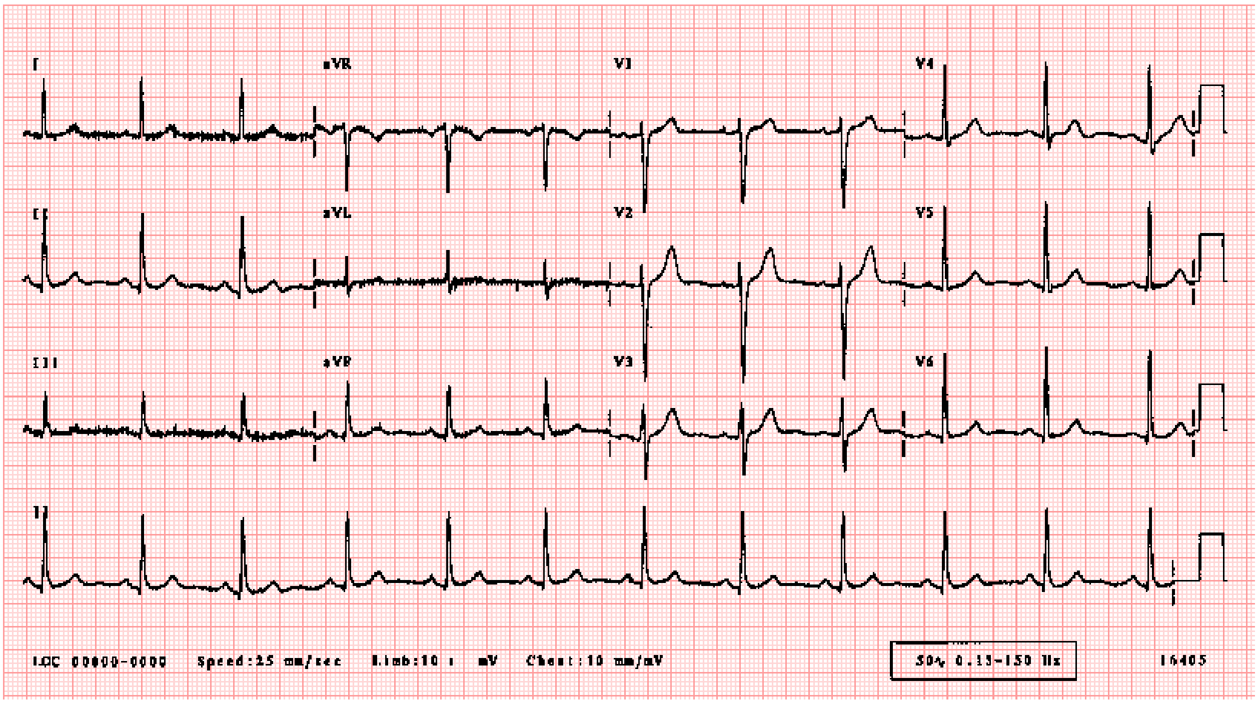

17. How would you manage the case?

Mark only one oval.

- ( ) General Anesthesia
- ( ) Spinal Anesthesia
- ( ) Other: ______________________

18. Do you need any other pre-operative examination? If yes, which one?

____

19. Any comment on Patient 5?

____

Thank you

The survey is finished! Thank you for participating!

20. Your mail (Optiional)

____

This content is neither created nor endorsed by Google.

Google Forms
