# Supplementary material for: Inattentional blindness in anesthesiology: A gorilla is worth one thousand words
Source: PLoS One. 2021 Sep 23;16(9):e0257508. doi: 10.1371/journal.pone.0257508 (PMC8459955; doi:10.1371/journal.pone.0257508)
Supplement: Attachment — Submitted filename: PAdova 18_07_2021.docx [file pone.0257508.s003.docx]

Padova 18/07/2021

Dear Editor,

Dear Reviewer,

We are submitting a revised version of our manuscript.

We hope that our manuscript meets the high standards of PLOS ONE and it is evaluable for publication!

Thank you for your consideration!

Alessandro De Cassai on behalf of all coauthors

**Editor**

**1. Please ensure that your manuscript meets PLOS ONE's style requirements, including those for file naming. The PLOS ONE style templates can be found at**

[**https://journals.plos.org/plosone/s/file?id=wjVg/PLOSOne_formatting_sample_main_body.pdf**](https://journals.plos.org/plosone/s/file?id=wjVg/PLOSOne_formatting_sample_main_body.pdf) **and**

[**https://journals.plos.org/plosone/s/file?id=ba62/PLOSOne_formatting_sample_title_authors_affiliations.pdf**](https://journals.plos.org/plosone/s/file?id=ba62/PLOSOne_formatting_sample_title_authors_affiliations.pdf)

We modified manuscript accordingly

**2. PLOS ONE does not copy edit accepted manuscripts (**[**https://journals.plos.org/plosone/s/criteria-for-publication#loc-5**](https://journals.plos.org/plosone/s/criteria-for-publication#loc-5)**). To that effect, please ensure that your submission is free of typos and grammatical errors.**

We read the manuscript a further time removing some typos. Thank you for pointing it out.

**3. We note that Figure 1 in your submission contain copyrighted images. All PLOS content is published under the Creative Commons Attribution License (CC BY 4.0), which means that the manuscript, images, and Supporting Information files will be freely available online, and any third party is permitted to access, download, copy, distribute, and use these materials in any way, even commercially, with proper attribution. For more information, see our copyright guidelines:** [**http://journals.plos.org/plosone/s/licenses-and-copyright**](http://journals.plos.org/plosone/s/licenses-and-copyright)**.**

**We require you to either (1) present written permission from the copyright holder to publish these figures specifically under the CC BY 4.0 license, or (2) remove the figures from your submission:**

**a. You may seek permission from the original copyright holder of Figure 1 to publish the content specifically under the CC BY 4.0 license.**

**We recommend that you contact the original copyright holder with the Content Permission Form (**[**http://journals.plos.org/plosone/s/file?id=7c09/content-permission-form.pdf**](http://journals.plos.org/plosone/s/file?id=7c09/content-permission-form.pdf)**) and the following text:**

**“I request permission for the open-access journal PLOS ONE to publish XXX under the Creative Commons Attribution License (CCAL) CC BY 4.0 (**[**http://creativecommons.org/licenses/by/4.0/**](http://creativecommons.org/licenses/by/4.0/)**). Please be aware that this license allows unrestricted use and distribution, even commercially, by third parties. Please reply and provide explicit written permission to publish XXX under a CC BY license and complete the attached form.”**

**Please upload the completed Content Permission Form or other proof of granted permissions as an "Other" file with your submission.**

**In the figure caption of the copyrighted figure, please include the following text: “Reprinted from [ref] under a CC BY license, with permission from [name of publisher], original copyright [original copyright year].”**

**b. If you are unable to obtain permission from the original copyright holder to publish these figures under the CC BY 4.0 license or if the copyright holder’s requirements are incompatible with the CC BY 4.0 license, please either i) remove the figure or ii) supply a replacement figure that complies with the CC BY 4.0 license. Please check copyright information on all replacement figures and update the figure caption with source information. If applicable, please specify in the figure caption text when a figure is similar but not identical to the original image and is therefore for illustrative purposes only.**

We were unable to obtain permission from the original copyright holder to publish under CC BY 4.0. For these reason we supply a replacement adding in the figure legend that the figure is similar but not identical to the original image and is therefore for illustrative purposes only.

**4. We note that Figure 2 in your submission contain [map/satellite] images which may be copyrighted. All PLOS content is published under the Creative Commons Attribution License (CC BY 4.0), which means that the manuscript, images, and Supporting Information files will be freely available online, and any third party is permitted to access, download, copy, distribute, and use these materials in any way, even commercially, with proper attribution. For these reasons, we cannot publish previously copyrighted maps or satellite images created using proprietary data, such as Google software (Google Maps, Street View, and Earth). For more information, see our copyright guidelines:** [**http://journals.plos.org/plosone/s/licenses-and-copyright**](http://journals.plos.org/plosone/s/licenses-and-copyright)**.**

**We require you to either (1) present written permission from the copyright holder to publish these figures specifically under the CC BY 4.0 license, or (2) remove the figures from your submission:**

**a. You may seek permission from the original copyright holder of Figure 2 to publish the content specifically under the CC BY 4.0 license.**

**We recommend that you contact the original copyright holder with the Content Permission Form (**[**http://journals.plos.org/plosone/s/file?id=7c09/content-permission-form.pdf**](http://journals.plos.org/plosone/s/file?id=7c09/content-permission-form.pdf)**) and the following text:**

**“I request permission for the open-access journal PLOS ONE to publish XXX under the Creative Commons Attribution License (CCAL) CC BY 4.0 (**[**http://creativecommons.org/licenses/by/4.0/**](http://creativecommons.org/licenses/by/4.0/)**). Please be aware that this license allows unrestricted use and distribution, even commercially, by third parties. Please reply and provide explicit written permission to publish XXX under a CC BY license and complete the attached form.”**

**Please upload the completed Content Permission Form or other proof of granted permissions as an "Other" file with your submission.**

**In the figure caption of the copyrighted figure, please include the following text: “Reprinted from [ref] under a CC BY license, with permission from [name of publisher], original copyright [original copyright year].”**

**b. If you are unable to obtain permission from the original copyright holder to publish these figures under the CC BY 4.0 license or if the copyright holder’s requirements are incompatible with the CC BY 4.0 license, please either i) remove the figure or ii) supply a replacement figure that complies with the CC BY 4.0 license. Please check copyright information on all replacement figures and update the figure caption with source information. If applicable, please specify in the figure caption text when a figure is similar but not identical to the original image and is therefore for illustrative purposes only.**

**The following resources for replacing copyrighted map figures may be helpful:**

**USGS National Map Viewer (public domain):** [**http://viewer.nationalmap.gov/viewer/**](http://viewer.nationalmap.gov/viewer/)

**The Gateway to Astronaut Photography of Earth (public domain):** [**http://eol.jsc.nasa.gov/sseop/clickmap/**](http://eol.jsc.nasa.gov/sseop/clickmap/)

**Maps at the CIA (public domain):** [**https://www.cia.gov/library/publications/the-world-factbook/index.html**](https://www.cia.gov/library/publications/the-world-factbook/index.html) **and** [**https://www.cia.gov/library/publications/cia-maps-publications/index.html**](https://www.cia.gov/library/publications/cia-maps-publications/index.html)

**NASA Earth Observatory (public domain):** [**http://earthobservatory.nasa.gov/**](http://earthobservatory.nasa.gov/)

**Landsat:** [**http://landsat.visibleearth.nasa.gov/**](http://landsat.visibleearth.nasa.gov/)

**USGS EROS (Earth Resources Observatory and Science (EROS) Center) (public domain):** [**http://eros.usgs.gov/#**](http://eros.usgs.gov/#)

**Natural Earth (public domain):** [**http://www.naturalearthdata.com/**](http://www.naturalearthdata.com/)

We are not sure who is the original copyright holder of the map we used. For this reason we recreate the map starting from an image with CC BY 4-0 license

**5. Please include captions for your Supporting Information files at the end of your manuscript, and update any in-text citations to match accordingly. Please see our Supporting Information guidelines for more information:** [**http://journals.plos.org/plosone/s/supporting-information**](http://journals.plos.org/plosone/s/supporting-information).

Done

**6. Please review your reference list to ensure that it is complete and correct. If you have cited papers that have been retracted, please include the rationale for doing so in the manuscript text, or remove these references and replace them with relevant current references. Any changes to the reference list should be mentioned in the rebuttal letter that accompanies your revised manuscript. If you need to cite a retracted article, indicate the article’s retracted status in the References list and also include a citation and full reference for the retraction notice.**

We reviewed the references, however, we were not able to see any retracted article among the articles we cited. However, if you believe some of papers we have cited has been retracted please let us know so we could remove it from reference list.

**7. I agree with the reviewer, this study injects some humour into a serious issue and adds novelty in a similar way to Christmas BMJ. I intend to accept this submission but I would like the reviewers to go away and expand the discussion to include semantically similar concepts to inattentional blindness in their discussion. They have cited the one previous simulation study but for the authors' own benefit and for the benefit of increased future impact, I think they could expand to discuss similar/relevant concepts such as situational awareness, as they have been studies in anaesthetics, peri-operative or critical care circumstances. Have some examples as follows-** [**https://blog.oup.com/2015/01/perceptual-errors-inattenional-blindness/**](https://blog.oup.com/2015/01/perceptual-errors-inattenional-blindness/) **and** [**https://pubs.asahq.org/anesthesiology/article/118/3/729/13561/Situation-Awareness-in-AnesthesiaConcept-and**](https://pubs.asahq.org/anesthesiology/article/118/3/729/13561/Situation-Awareness-in-AnesthesiaConcept-and) **(NB: these are not self-references). Situational blindness is a critical concept in human factors studies and I also think it's something you'll find has highly relevant military and aviation links. I look forward to reading the response, the intention is not to get you to completely rework the paper, just to consider adding some generalisability and additional value. Kind regards,**

We tried to expand the discussion adding value to the overall manuscript. Thank you for your comment.

**Reviewers' comments:**

**Comments to the Author**

**Reviewer #1: The authors present a wonderful piece of research: they raise attention for a very serious topic with a highly entertaining method, and thus succeed in making the reader both think and smile.**

**Inattentional blindness is, as pointed out in their discussion, a major topic for health care providers, especially in but definitely not limited to perioperative care. The authors confronted anaesthesiologists with medical cases, in which a gorilla face was hidden in a chest-x-ray. Only one third spotted the gorilla.**

**Of course, the study can be criticized, as biases in the selection of the sample and the response rates are not completely transparent, (as the authors mention in their discussion) but this does not weaken the fundamental statement that a large proportion of experienced clinicians were not aware of the gross conspicuity. I congratulate the authors for their work! Thank you!**

We would like to thank the reviewer for his kind words
