# Supplementary material for: Inattentional blindness in anesthesiology: A gorilla is worth one thousand words
Source: PLoS One. 2021 Sep 23;16(9):e0257508. doi: 10.1371/journal.pone.0257508 (PMC8459955; doi:10.1371/journal.pone.0257508)
Supplement: Attachment — Submitted filename: Padova 01_09_2021.docx [file pone.0257508.s004.docx]

Padova 01/09/2021

Dear Editor,

Dear Reviewers,

Thank you for your time and effort in improving our manuscript. We deeply appreciate it.

We tried to respond to your concerns and to modify the manuscript accordingly

We hope our manuscript is suitable for publication in your prestigious journal!

Alessandro De Cassai on behalf of all coauthors

**Reviewer #1: The authors present a revised version of their manuscript. They included satisfying responses all issues raised.**

Reviewer #1 We would like to thanks Reviewer#1 for his kind words.

**Reviewer #2:**

**Q)Instead of a general remark (ever-growing attention is deemed necessary to improve patient safety.), more specific suggestions should be provided. The conclusion consists of only one sentence**

A)Thank you for your comment. We added the following to the conclusion section:

“Inattentional blindness is a common and unavoidable phenomenon in anesthesia. An ever-growing attention is deemed necessary to improve patient safety; to achieve this objective several strategies should be adopted such as an increased use of standardized protocols, promoting automation based strategies to reduce human error when performing repetitive tasks and discouraging evaluation of multiple consecutive patients in the same work shifts independently of the associated complexity.”

**Q)This study analyzed only incidence rates. How about diagnostic accuracy for each case? For each simulated case, explain in detail where to look at for a typical diagnosis.**

A)Dear Reviewer, there was not a “correct answer”. We tried to explain this adding the following paragraph to the method section:

“Each case proposed a healthy patient or one with minor health problems undergoing non-major general surgery which did not present any particular concern or challenge related to the anesthetic management. Given the above, we would like to highlight that there cannot be a universally accepted correct answer by the respondents (e.g. spinal anesthesia compared to general anesthesia).

We decided to focus our survey on these simple cases in order to both increase respondent confidence with patient management irrespective of the personal experience gained as anesthesiologists and to investigate inattentional blindness during routine work.”

**Q)Each section should consist of paragraphs, but some sections do not have paragraphs. In addition, some sentences have grammatical errors.**

A)Thank you for noticing it, we corrected some typos in the text.

If the reviewer thinks more paragraph titles are appropriate we kindly ask to indicate to us which one we should add.

**Q)When simulated tasks are used, it is essential to ensure their validity. Discuss their validity.**

**1. Are simulated tasks valid in terms of image size, resolution, visual angle, task time, etc?. How seriously would the respondent consider each case?. How confidently would the respondent answer each question?. How similar were the five cases to the actual cases in terms of workload?**

A)Validity is discussed in the pretesting section of the manuscript:

In order to respond to the second part of your question we added the following to the method section

“Each case proposed a healthy patient or one with minor health problems undergoing non-major general surgery which did not present any particular concern or challenge related to the anesthetic management. Given the above, we would like to highlight that there cannot be a universally accepted correct answer by the respondents (e.g. spinal anesthesia compared to general anesthesia).

We decided to focus our survey on these simple cases in order to both increase respondent confidence with patient management irrespective of the personal experience gained as anesthesiologists and to investigate inattentional blindness during routine work.”

Moreover, we added the following in the limitation section

Third, we recognize that given the nature of the study (a survey distributed via social network) it is not possible to determine how seriously the respondents assessed each case.

Describe how the developed survey meets the BRUSO criteria.

We added the following in the method section

“In order to achieve this objective we built our questionnaire keeping both the questionnaire and each question as short as possible in order to reduce the rate of partially completed questionnaires, we tried to avoid misleading or ambiguous terms and we asked only relevant questions.”

If laboratory tests, chest radiography and electrocardiogram are the same for the first several cases, without pathological findings, it is very unlikely to pay attention to these data for the remaining cases, thus more likely to miss the gorilla.

Thank you for pointing out this point. We added the following in the discussion section:

In our study anesthesiologists were asked to plan anesthesia management for five simulated cases. The proposed cases did not pose any particular challenge to the respondents because patients were healthy or with only minor health issues. Most patients undergoing anesthesia for minor surgery do not pose particular concerns to anesthesiologists, however the repetitive task of evaluating these patients could lead to lower attention threshold if the anesthesiologist is required to evaluate several of these patients consecutively in a brief time.

How did you conclude whether the gorilla was (was not) spotted by respondents? I don't see a direct question on this.

Added the following in the method section

“ For the main objective of this study, the question “Any comment on Patient 5?” was used to

calculate the gorilla detection rate. However, we decided to consider the identification successful also if the physician incorrectly named the animal or labelled the cardiac shadow in the chest radiograph as “not normal”.”

**Q)The abstract should be rewritten.**

**1. The abstract should be one paragraph without any subsections.**

Dear Reviewer, in many PLOSone article subsections are used. We would like to keep the subsections, however, if editor request it we will remove it.

**2. An online survey was spread on Facebook (Facebook, Inc., Menlo Park, California, United States) between May 1, 2021 and May 31, 2021: Too much information is provided.**

Taken

**3. The survey consisted of simulated cases.: More information is needed. (The survey consisted of five simulated cases.)**

Taken

**4. There are no meaningful findings/implications.**

Taken

**Q)In the introduction, the nature of anaesthesia-related tasks should be described in detail for readers without medical information. For example, what types of attention (focused, selective, divided, and sustained) are relevant to anaesthesia-related tasks ? How many and what data channels are involved? and what is a typical procedure involved in diagnosis?**

A) Added the following in the introduction:

Anaesthesia is a complex discipline which requires a high level of attention and is characterized by periods of intense activity while managing multiple tasks at the same time and periods with a lower workload demand. The lower workload periods may occupy substantial parts of the routine cases (up to 40%) being associated with lower cognitive and physical demand [slagle]. However, during the intense activity phase, the anesthesiologist has to pay different types of attention to ensure patient safety. The required attention types are focused (e.g.vital signs alarms), divided (e.g. evaluating at the same time patient’s vital parameters, surgical field and the equipment such as ventilator at the same time), selective (e.g. selecting and choosing the important input from the multiple parameter monitor suppressing irrelevant or distracting information) and sustained (e.g. patient undergoing emergent major surgery for several hours and requiring hemodynamic and respiratory management throughout the duration of the surgery).

**Q)The statistical analysis section should be described more in detail.**

We recognize that the statistical analysis section is quite small. However, it is the statistical analysis we actually used. We do not believe that increasing the paragraph would be helpful to the readers to replicate our study.

**Reviewer #3: Inattentional blindness is a serious concern that is somewhat lacking attention in the public and in serious workplaces. I agree with the previous reviewer and editor, the authors do a good job bringing light to this topic using humility that will attract a wide audience to a serious topic. I also agree that there are some pitfalls to this paper and study; however, I agree with the previous reviewer that they do not affect the overall statement of the paper.**

We would like to thank the reviewer for his comment.

**Despite that, I do have a few minor comments regarding grammar:**

**1) Although not significant, the rate at which the gorilla was spotted did trend towards decreasing with experience. Please be sure to mention this in your discussion in the topic regarding age and inattentional blindness.**

Dear reviewer, we would be happy to find a statistical significance (because this finding would agree with the previous literature). However, it was not. We believe that stating that we “found a trend towards decrease with experience” would be not totally valid. And if reviewer and editor agree we would prefer to leave the sentence in its original form.

**2) Abstract, Materials and Methods - please replace "spreaded" with spread or distributed.**

Taken

**3) Page 7 (Methods, Population of Interest and Survey Distribution Plan - please revise "we initiated to spread the invite to respond to the survey" to "We spread an invite to the survey" or "We began distribution of the survey invitation" or equivalent**

Taken

**4) Page 8 (Results, paragraph 1) - there are two references to figure 2. Please remove one.**

Taken

**5) Page 11 (Discussion, sentence 1) - remove that from "exists in the anesthesia field and that may be far more common than generally thought"**

Taken

**6) Page 13 (discussion) - remove the new line following "our study has some limitations that we would like to discuss"**

Taken

**7) Page 14 (discussion) - reword "using as example a wave analysis model"**

Taken
